# In Silico, In Vitro and In Vivo Analysis of Tanshinone IIA and Cryptotanshinone from *Salvia miltiorrhiza* as Modulators of Cyclooxygenase-2/mPGES-1/Endothelial Prostaglandin EP3 Pathway

**DOI:** 10.3390/biom12010099

**Published:** 2022-01-07

**Authors:** Anella Saviano, Simona De Vita, Maria Giovanna Chini, Noemi Marigliano, Gianluigi Lauro, Gian Marco Casillo, Federica Raucci, Maria Iorizzi, Robert Klaus Hofstetter, Katrin Fischer, Andreas Koeberle, Oliver Werz, Francesco Maione, Giuseppe Bifulco

**Affiliations:** 1 *ImmunoPharmaLab*, Department of Pharmacy, School of Medicine and Surgery, University of Naples Federico II, Via Domenico Montesano 49, 80131 Naples, Italy; nella.1993@hotmail.it (A.S.); noemi.marigliano@outlook.com (N.M.); gianmarcocasillo@virgilio.it (G.M.C.); federica.raucci@unina.it (F.R.); 2 Department of Pharmacy, University of Salerno, Via Giovanni Paolo II 132, 84084 Fisciano, Italy; sdevita@unisa.it (S.D.V.); glauro@unisa.it (G.L.); 3 Department of Biosciences and Territory, University of Molise, Contrada Fonte Lappone, Pesche, 86090 Isernia, Italy; mariagiovanna.chini@unimol.it (M.G.C.); iorizzi@unimol.it (M.I.); 4 Department of Pharmaceutical/Medicinal Chemistry, Institute of Pharmacy, Friedrich-Schiller-University Jena, Philosophenweg 14, 07743 Jena, Germany; robert.klaus.hofstetter@uni-jena.de (R.K.H.); katrin.fischer.1@uni-jena.de (K.F.); oliver.werz@uni-jena.de (O.W.); 5 Michael Popp Institute, University of Innsbruck, Mitterweg 24, 6020 Innsbruck, Austria; Andreas.Koeberle@uibk.ac.at

**Keywords:** docking, EPs, mPGES-1, platelet aggregation, tanshinones

## Abstract

Tanshinone IIA (TIIA) and cryptotanshinone (CRY) from *Salvia miltiorrhiza* Bunge were investigated for their inhibitory activity against the cyclooxygenase-2 (COX-2)/microsomal prostaglandin E synthase-1 (mPGES-1)/endothelial prostaglandin 3 (EP3) pathway using in silico, in vitro, in vivo, and ex vivo assays. From the analysis of the docking poses, both diterpenoids were able to interact significantly with COX-2, 5-lipoxygenase (5-LO), platelet-activating factor receptor (PAFR), and mPGES-1. This evidence was further corroborated by data obtained from a cell-free assay, where CRY displayed a significant inhibitory potency against mPGES-1 (IC_50_ = 1.9 ± 0.4 µM) and 5-LO (IC_50_ = 7.1 µM), while TIIA showed no relevant inhibition of these targets. This was consistent with their activity to increase mice bleeding time (CRY: 2.44 ± 0.13 min, *p* ≤ 0.001; TIIA: 2.07 ± 0.17 min *p* ≤ 0.01) and with the capability to modulate mouse clot retraction (CRY: 0.048 ± 0.011 g, *p* ≤ 0.01; TIIA: 0.068 ± 0.009 g, *p* ≤ 0.05). For the first time, our results show that TIIA and, in particular, CRY are able to interact significantly with the key proteins involved not only in the onset of inflammation but also in platelet activity (and hyper-reactivity). Future preclinical and clinical investigations, together with this evidence, could provide the scientific basis to consider these compounds as an alternative therapeutic approach for thrombotic- and thromboembolic-based diseases.

## 1. Introduction

Inflammation is a complex defense mechanism characterized by leukocyte extravasation from the vasculature to local tissue damage due to injurious and noxious agents/stimuli [1,2]. Neutrophils dominate the initial influx of leukocytes, followed by monocytes and macrophages. These events are correlated with a transient increase of pro-inflammatory mediators, including cytokines, chemokines, prostaglandins (PGs), and leukotrienes (LTs) [3,4,5]. Moreover, the development of inflammatory conditions is often associated with cardiovascular alterations of platelet activity (and hyper-reactivity) and, to a lesser extent, thrombotic and thromboembolic complications [6,7,8].

PGs and LTs are powerful bioactive lipid mediators that are involved in the onset of inflammation and in numerous homeostatic functions [9,10]. The biosynthesis of PGs is initialized by cyclooxygenases (COXs) isoenzymes (COX-1 and COX-2) that convert arachidonic acid to PGH_2_ [11], which is subsequently isomerized to prostaglandin (PG) E_2_ by three different PGE_2_ synthases situated downstream of the COXs [12]. The cytosolic prostaglandin E_2_ synthase (cPGES) and the microsomal prostaglandin E_2_ synthase (mPGES)-2 are constitutive enzymes, whereas mPGES-1 is an inducible isoform [13,14,15]. Consistently, the upregulation of mPGES-1 expression and the involvement of the COX-2/mPGES-1/PGE_2_ cascade in terms of PGs production has been extensively reported in pathological settings in which PGE_2_ is implicated, such as fever, pain, and inflammatory-based disease [16,17,18].

Several lines of evidence also indicate that mPGES-1 activity is detrimental for platelet aggregation [19,20,21], thrombosis, and hemostasis [22,23,24] and that PGE_2_, thromboxane (TXA), and matrix metalloproteinase (MMPs) are expressed on platelets in both resting and activating conditions [25,26,27].

PGE_2_ acts on four specific G-protein-coupled receptors (GPCR) subtypes, named endothelial prostaglandin 1–4 (EP1–4), mediating different biological functions, and exhibiting a biphasic effect on platelet aggregation [28]. Specifically, EP2, EP3, and EP4 are all expressed on platelets, and the expression level of EP3 is much higher than EP2 and EP4. Functionally, EP3 mediates the pro-aggregatory effect of PGE_2_-dependent [29,30,31]; whereas EP2 and EP4 signaling mediate anti-aggregatory effects.

**Figure 1:**
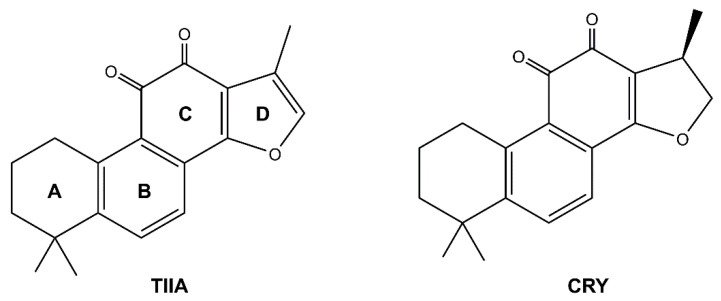
Chemical structure of TIIA and CRY.

Considerable interest has been expressed in natural products and their bioactive components that exert antiplatelet activities to reduce thrombotic risk and related cardiovascular complications [32,33,34]. In this context, tanshinone IIA (TIIA) and cryptotanshinone (CRY), the main lipophilic components from the rhizome of *Salvia miltiorrhiza* Bunge, have been widely used for the effective treatment of atherothrombosis in traditional Chinese medicine (Figure 1) [35,36].

Until now, the molecular mechanisms of action of these two diterpenoids on platelets are only partially known. We have previously demonstrated that TIIA and CRY are able to inhibit rat platelet aggregation via the modulation of tubulin acetylation and the inhibition of extracellular signal-regulated kinases-2 (ERK-2) phosphorylation [37] and that their effects were mediated by the interaction with the G_i_-coupled P2Y12 receptor [38]. On these bases, their role on key targets involved in thrombogenic processes was investigated using a combined in silico, in vitro, in vivo, and ex-vivo approach to provide further insights into the biological activities of these natural compounds.

## 2. Materials and Methods

### 2.1. Computational Details

#### 2.1.1. Input File Preparation

The chemical structures of TIIA and CRY were built using the graphical interface of the Schrödinger Suite [39] and processed with LigPrep [40] to adjust the partial charges and the protonation state of the compounds. The following structures were collected from the Protein Data Bank [41]: secreted phospholipase A_2_ (sPLA_2_) (PDB: 3U8H [42]), COX-1 (PDB ID: 3KK6 [43]), COX-2 (PDB ID: 5KIR [44]), 5-lipoxygenase (5-LO) (PDB ID: 3O8Y [45]), mPGES-1 (PDB ID: 5K0I [46]), platelet-activating factor receptor (PAFR) (PDB ID: 5ZKQ [47]), EP3 (PDB ID: 6AK3 [48]), EP4 (PDB ID: 5YWY [49]), MMP-1 (PDB ID: 966C [50]), MMP2 (PDB ID: 1HOV [51]). The structures were prepared with the Protein Preparation Wizard [52,53], which determines the protonation state of each atom, adjusts the bond orders, and optimizes the intramolecular hydrogen bond network. The centroid of each ligand (where available) was used as a guide to build the necessary molecular docking grid. Due to the lack of a co-crystallized molecule in the 5-LO structure, the protocol illustrated in previous work [54] was followed. In detail, the centroid of key binding site residues (Phe177, Tyr181, His367, Leu368, His372, Leu373, Ile406, Leu414, His550, Asn554, Leu607, Ile673) was considered as the center and the inner box was extended for 10 Å and the outer box for 20 Å in the three spatial dimensions.

#### 2.1.2. Molecular Docking

For all the targets considered, except for 5-LO, a semiflexible molecular docking approach was followed using the software Glide [55,56,57,58] in Extra Precision (XP) mode. In detail, 10,000 poses were initially generated for each ligand and evaluated to select 800 conformations to enter the minimization step with an energetic cutoff of 0.15 kcal/mol. After that, a maximum of 15 poses was kept for each ligand for the visual inspection.

Concerning 5-LO, an Induced Fit approach [59,60,61,62] was used to mimic the high flexibility of the binding pocket residues [63]. We used the extended sampling protocol keeping all the residues flexible within 5 Å from the ligand and generating up to 80 poses for each molecule.

### 2.2. Cell-Free mPGES-1 Activity Assay

A549 cells were cultured and treated as described elsewhere [64,65]. In brief, to induce the expression of mPGES-1, cells were incubated for 72 h with 1 ng/mL of interleukin-1β (IL-1β). Afterward, cells were harvested with trypsin/EDTA, collected, sonicated, and centrifuged at 4 °C at 10,000× *g* for 10 min and then at 174,000× *g* for 1 h. The pellet containing the microsomal fraction was resuspended in 1 mL of buffer containing 0.1 M potassium phosphate buffer (pH 7.4), 1 mM phenylmethanesulfonyl fluoride, 60 μg/mL soybean trypsin inhibitor, 1 μg/mL leupeptin, 2.5 mM glutathione (GSH), and 250 mM sucrose. After the protein concentration was determined, the fraction was diluted in phosphate buffer 1.0 M (pH 7.4) with the addition of 2.5 mM GSH. The compounds and DMSO (as control) were added to the microsomal fraction and incubated for 15 min at 4 °C. The addition of 20 μM PGH_2_ started the enzymatic conversion, which was stopped after 1 min by adding 100 μL of stop solution (40 mM FeCl_2_, 80 mM citric acid, and 10 μM RP-HPLC 11β-PGE_2_). The PGE_2_ produced was extracted by solid-phase extraction and analyzed with RP-HPLC.

### 2.3. Cell-Free 5-LO, COX-1 and COX-2 Assays

5-LO was expressed in *E. coli* BL21 (DE3) transformed with the pT3-5-LO plasmid and purified by affinity chromatography on an ATP-agarose column [66]. The isolated enzyme was used immediately for 5-LO activity assays as described in detail in [67]. Briefly, 0.5 µg of 5-LO in PBS pH 7.4 containing EDTA (1 mM) were pre-incubated with TIIA, CRY, or vehicle (0.1% DMSO). After 15 min, samples were pre-warmed for 30 s at 37 °C before adding 2 mM CaCl_2_ plus 20 µM AA to start 5-LO product formation. After 10 min at 37 °C, an equal volume of ice-cold methanol was added to quench the reaction, and the formed 5-LO products were extracted by adding 500 µL acidified PBS and 200 ng of internal PGB_1_ standard before solid-phase extraction. Eluates were analyzed for 5-LO products (tr-LTB_4_ and 5-H(P)ETE) by RP-HPLC using a C-18 Radial-PAK column (Waters, Eschborn, Germany).

COXs inhibition was assayed using purified ovine COX-1 and recombinant human COX-2 as described in [68]. Briefly, COX-1 (50 U/mL) or COX-2 (20 U/mL) were diluted in Tris buffer (100 mM, pH 8) containing glutathione (5 mM), EDTA (100 µM), and hemoglobin (5 µM). After pre-incubation with TIIA, CRY, or vehicle (0.1% DMSO) for 5 min at room temperature (RT), the samples were pre-warmed for 30 sec at 37 °C, and the reactions were started with 5 µM AA (COX-1) or 2 µM AA (COX-2). After 5 min at 37 °C, the reactions were stopped by the addition of an equal volume of ice-cold methanol. Solid-phase extraction was performed as described above, and COXs product formation was determined by analysis of 12-hydroxyheptadecatrienoic acid (12-HHT) formation.

### 2.4. Materials

TIIA, CRY (≥97% and ≥98%, respectively, HPLC), and acetylsalicylic acid (ASA) were obtained from Sigma-Aldrich Co. (Milan, Italy). For western blot analysis, anti-PTGER2 was obtained from Origene (Rockville, MD, USA), anti-PTGER3 and anti-PTGER4 from Proteintech (Manchester, UK), and anti-alpha tubulin from SICGEN (Cantanhede, Portugal). HRP-conjugated IgG secondary antibodies were purchased from Dako (Copenhagen, Denmark). Unless otherwise stated, all the other reagents were from BioCell (Milan, Italy).

### 2.5. Animals

CD-1 male mice (12 weeks of age) were purchased from Charles River (Milan, Italy) and kept in an animal care facility under controlled temperature, humidity, and on a 12/12 light: dark cycle, with ad libitum access to water and a standard laboratory chow diet. All animal care and experimental procedures complied with the international and national law and policies and were approved (authorization number: 545/2021-PR) by the Italian Ministry of Health (EU Directive 2010/63/EU for animal experiments, and the Basel declaration including the 3Rs concept) [69,70]. All procedures were carried out to minimize the number of animals used (*n* = 7 per group) and their suffering.

### 2.6. In Vivo Bleeding Time 

For the in vivo model, a well-established method of bleeding time was used [71]. Mice were anesthetized intraperitoneally (i.p.) with a mixture of ketamine (75 mg/kg) and xylazine (10 mg/kg), and the tail was pre-warmed for 3 min in a 0.9% saline solution at 37 °C 1 h after the i.p. injection of TIIA and CRY (both at the dose of 10 mg/kg), ASA (10 mg/kg), and their vehicles (8% methanol in saline and 1% CMC in saline, for tanshinones and ASA, respectively). The bleeding was induced by a precise incision of the mouse tail 5 mm from the tip. The distal portion of the tail (3 cm) was immersed vertically into the 0.9% saline solution at 37 °C. Blood flowing from the incision was carefully monitored, and the time to cessation of bleeding was recorded as the bleeding time. Bleeding cessation was the time when the flow of blood stopped.

### 2.7. Ex Vivo Clot Retraction

The protocol proposed by Law et al. with slight modifications was adopted [72]. Briefly, non-anticoagulated blood samples, obtained by intracardiac puncture (300 μL) were transferred into a Microvette^®^ 300 Z (Sarstedt, Verona, Italy) containing a clotting activator and incubated at room temperature for 2 h to get clot formation. Thereafter, clots were collected and weighed (g), and residual serum volumes (μL) were pipetted as an indirect value of reaction [73].

### 2.8. Western Blot Analysis

Whole clots, previously homogenated, were subjected to SDS-PAGE (10% gel) using standard protocols as previously described [74,75]. The proteins (35 μg) were transferred to nitrocellulose membrane (0.2 µm nitrocellulose membrane, Trans-Blot^®^ TurboTM, Transfer Pack, Bio-Rad Laboratories, Hercules, CA, USA) in the transfer buffer (25 mM Tris–HCl (pH 7.4) containing 192 mM glycine and 20% *v*/*v* methanol) at 400 mA for 2 h. The membranes were saturated by incubation for 2 h at RT with non-fat dry milk (5% *w*/*v*) in PBS supplemented with 0.1% (*v*/*v*) Tween 20 (PBS-T), and then incubated with 1:1000 dilution of primary antibodies overnight at 4 °C with rabbit polyclonal anti-PTGER2 (TA351556), rabbit polyclonal anti-PTGER3 (24761-I-AP), rabbit polyclonal anti-PTGER4 (66921-1-Ig), goat polyclonal anti- alpha tubulin (AB0134-200), and then washed 3 times with PBS-T. In all cases, blots were then incubated with a 1:3000 dilution of related horseradish peroxidase-conjugated secondary antibody for 2 h at RT and finally washed 3 times with PBS-T. Protein bands were detected by using the enhanced chemiluminescence method (ClarityTM Western ECL Substrate, Bio-Rad Laboratories, Hercules, CA, USA) and Image Quant 400 GE Healthcare software (GE Healthcare, Milan, Italy). Finally, protein bands were quantified using the GS 800 imaging densitometer software (Biorad, Milan, Italy) and normalized with respective tubulin. 

### 2.9. Data and Statistical Analysis

The data and statistical analysis in this study comply with the international recommendations on experimental design and analysis in pharmacology [76] and data sharing and presentation in preclinical pharmacology [77,78]. The results obtained were expressed as the mean ± SD. Statistical analyses were performed by using one- or two-way ANOVA followed by Bonferroni testing when comparing more than two groups. GraphPad Prism 8.0 software (San Diego, CA, USA) was used for analysis. Data were considered statistically significant when a value of *p* ≤ 0.05 was achieved.

## 3. Results

### 3.1. Molecular Docking

The chemical structure of the secondary metabolites considered in this work is reported in Figure 1. Both these lipophilic compounds are diterpenoid containing a tetrahydronaphthalene moiety (rings A and B) fused to an *ortho*-quinone (ring C) and a furan or dihydrofuran (ring D) for TIIA and CRY, respectively [79]. The small difference in ring D between the two secondary metabolites makes the TIIA structure planar and rigid, preventing the ring from bending to accommodate inside the pocket, and form additional interactions similar to COX-2, and MMP-2 (*vide infra*).

**Figure 2:**
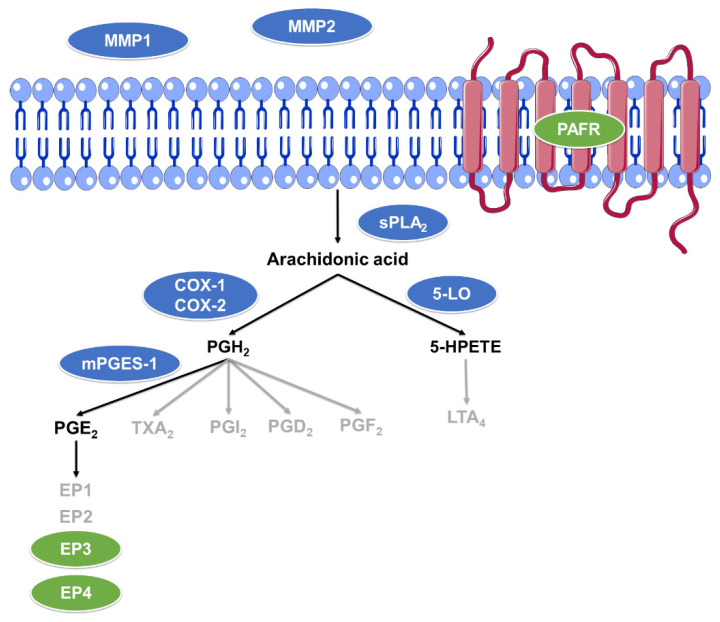
Inflammatory and aggregation pathways analyzed in this work. The elements excluded from the analysis are in gray, enzymes are enclosed in blue ovals, and receptors in green.

The first step to hypothesize a potential anti-inflammatory and/or anti-aggregant activity of the two tanshinones, TIIA and CRY, was performing molecular docking experiments on some relevant targets involved in these pathways. For this purpose, the following proteins were selected: sPLA_2_, COX-1, COX-2, 5-LO, mPGES-1, EP3, EP4, PAFR, MMP-1, and MMP-2 (Figure 2).

The structures were downloaded from the Protein Data Bank (https://www.rcsb.org/, accessed on 8 June 2020) [41,80] and cleaned from unnecessary elements like solvent molecules, ions, etc. Then, the proteins were prepared using the Protein Preparation Wizard [81], which corrects common structural errors in the protein (bond orders, protonation states, partial charges, etc.) and optimizes the intramolecular hydrogen bond network. Except for 5-LO, for which the procedures illustrated elsewhere were followed [82], all the proteins contained a co-crystallized molecule that was used to build the necessary grids for the subsequent molecular docking experiments. With this aim, the software Glide [55,56,57,58] was employed, and the resulting poses were visually inspected to determine the efficiency of the protein-ligand interactions. Moreover, a re-docking of the co-crystallized ligand was carried out to provide an energetic benchmark to estimate the binding of the two natural products; the chosen poses with the corresponding RMSD values from the co-crystallized ligand are reported in Figure S5 (Supplementary Material).

Based on the interactions made by the co-crystallized molecule and the information available [47,48,49,50,51,82,83,84,85,86,87,88,89,90,91], a set of key residues was identified for each target (Table 1). For the Ballesteros–Weinstein numbering scheme of PAFR, EP3, and EP4 please refer to Supporting Information.

**Figure 3:**
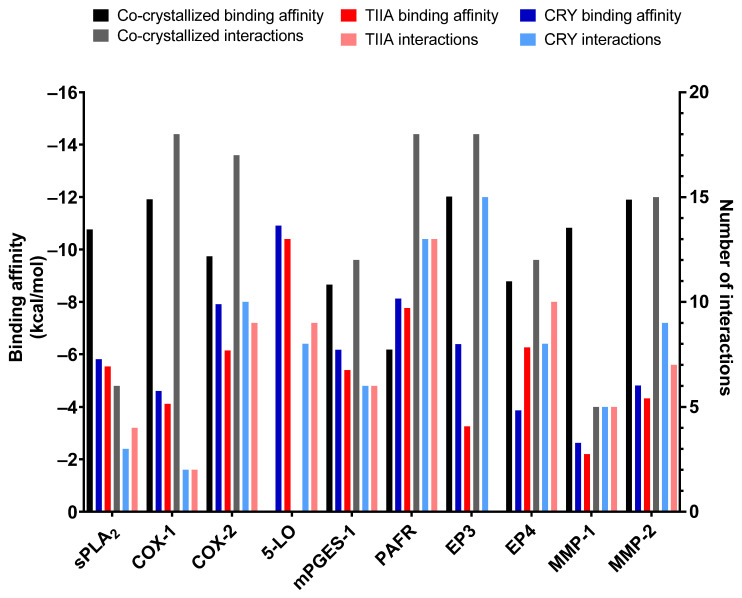
Binding energy and number of interactions made by the co-crystallized ligand (if available), TIIA, and CRY with each target.

From the analysis of the molecular docking poses, some interesting data emerged. For CRY, 5-LO and PAFR were the targets that showed the best results, in terms of binding affinity and number of interactions with the key residues (listed in Table 1). In the remaining cases, the two molecules showed comparable results except for COX-2 and EP3, where CRY performed slightly better than the counterpart, probably due to the higher mobility of the methyl moiety on ring D that favors its accommodation in the binding pocket instead of keeping it in a rigid position (Figure 3). A detailed analysis of the molecular docking results is reported below.

#### 3.1.1. sPLA_2_

The sPLA_2_ hydrolyzes arachidonic acid contained in the cell membrane, making it available to be transformed into a wide range of lipid mediators. It is a Ca^2+^-dependent enzyme, and the cation is responsible for the coordination of the glycerophospholipids before hydrolyzation [92]. The catalytic triad is represented by His47, Asp48, Asp89, and the Ca^2+^ ion [93]; the latter, though, is not strictly required for the binding of inhibitors [92]. Other important residues are those forming the hydrophobic cavity: Ile2, Leu5, Ala6, Val9, Pro17, Ile18, and Met21 [87].

**Figure 4:**
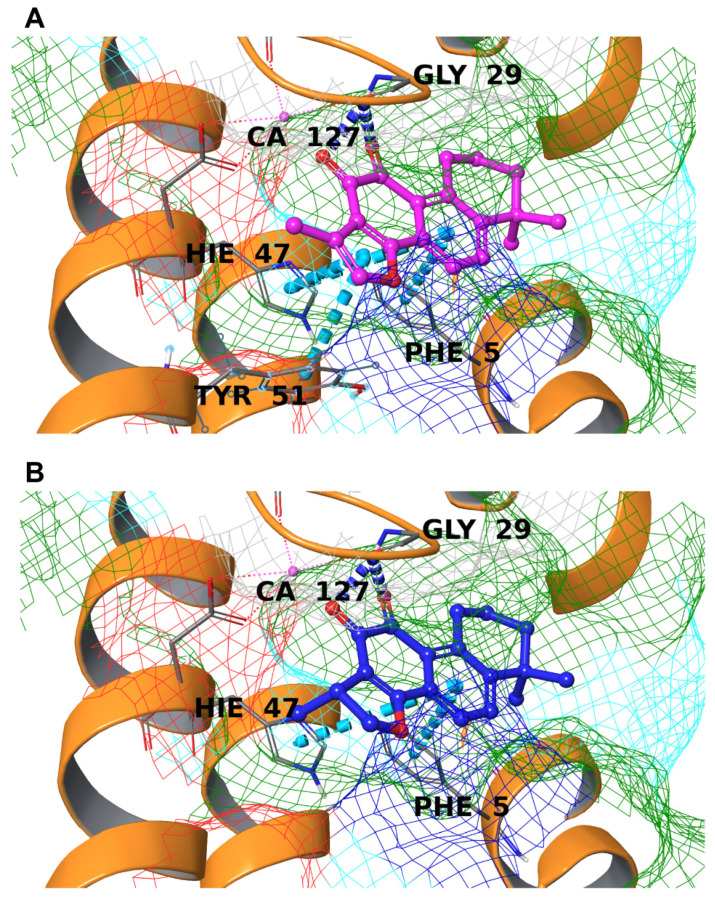
Binding mode of TIIA (**A**) and CRY (**B**) in the binding pocket of sPLA_2_. Hydrogen bonds are depicted as blue dotted lines, π-π stackings are represented as cyan dotted lines, and the calcium atom is represented as a fuchsia sphere and labeled. The molecular surface of the binding site is shown and colored according to residue property: green for hydrophobic residues, cyan for polar ones, blue for positively charged amino acids, and red for negatively charged ones. The interacting residues are labeled in black with HIE representing the ε-nitrogen protonated histidine residue.

Both compounds showed promising binding to the key amino acids highlighted above (Table 1), with very small differences in the calculated affinity and the three-dimensional arrangement inside the pocket. In detail, they form interactions with Gly29 (double hydrogen bond with the carbonyl groups) and His47 (π-π stacking), which are part of the catalytic triad, and additional contacts with Phe5 (both) and Tyr51 (only TIIA). Moreover, the lipophilic moiety is in perfect contact with the hydrophobic cavity surface (Figure 4). These considerations suggested the biological effect of these molecules on sPLA_2_. 

#### 3.1.2. COX-1 and COX-2

The substrate/inhibitor binding sites of COX-1 and COX-2 are notoriously very similar, with only an Ile/Val substitution in position 523 [94]. Each monomer consists of three different domains: the epidermal growth factor (EGF) domain, the membrane-binding domain, and the catalytic domain containing the catalytic triad Arg120, Tyr355, and Glu524. Among these domains, the catalytic triad represents the main target for non-steroidal anti-inflammatory drugs (NSAID) [95] and, particularly, Arg120, Tyr355, Tyr385, and Arg513 are essential for inhibitory activity [95,96]. The two co-crystallized molecules (celecoxib and rofecoxib, respectively, for COX-1 and COX-2) form a high number of interactions with the target due to their chemical structure, reflecting their excellent biological performance [97,98].

**Figure 5:**
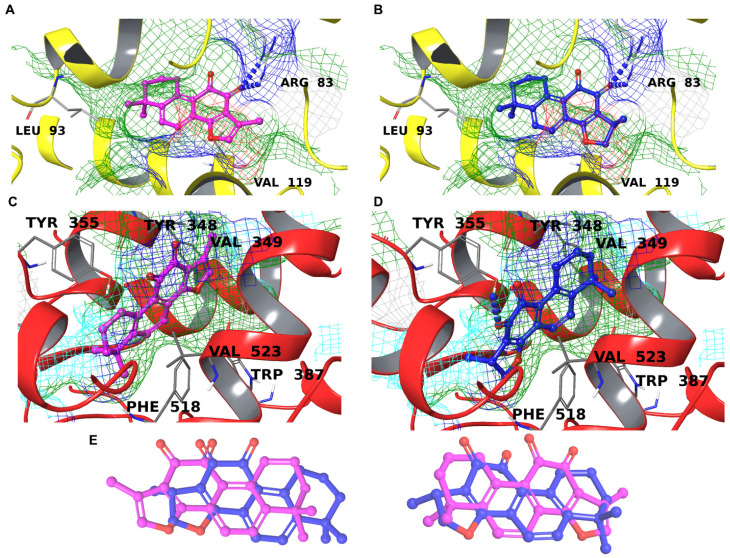
Binding mode of TIIA (magenta) and CRY (blue) inside the binding pockets of COX-1 (yellow ribbons) and COX-2 (red ribbons) (**A**–**D**). Hydrogen bonds are depicted as blue dotted lines. The molecular surface of the binding site is shown and colored according to residue property: green for hydrophobic residues, cyan for polar ones, blue for positively charged amino acids, and red for negatively charged ones. The important residues of the binding site are labeled in black. (**E**) Superimposition of the binding mode TIIA and CRY inside the binding pocket of COX-1 (right) and COX-2 (left).

The binding mode of TIIA and CRY was very similar in the case of COX-1; they both form a double hydrogen bond with Arg83 and make hydrophobic connections with Leu93 and Val119 (Figure 5A,B). Both molecules were inserted well in the binding site (delimited by a mesh colored according to the residue property) and made several contacts with the extended hydrophobic surface. Concerning COX-2, CRY formed a hydrogen bond with Tyr355 that, as mentioned above, is one of the key residues involved in the inhibition of this enzyme (Figure 5D), while TIIA interacted with the binding site only by hydrophobic contacts (Figure 5C), explaining the better predicted binding affinity of CRY with respect to TIIA. This difference between the two targets was due to the opposite binding pose of TIIA with respect to CRY (Figure 5E). The two ligands were almost superposed in the COX-1 binding site while their binding mode is the opposite in the case of COX-2, allowing only the carbonyl group of CRY to form a hydrogen bond with the OH group of Tyr355.

#### 3.1.3. 5-LO

The activity of 5-LO can be inhibited by four different types of molecules: redox, non-redox, competitive, and iron-chelating compounds [99,100,101]. The competitive inhibitors, in particular, may interact with the arachidonic acid binding pocket or with a putative allosteric site [101]. Considering the chemical nature of TIIA and CRY, it can be hypothesized that these may act as “canonical” competitive inhibitors (i.e., that interfere with the arachidonic acid binding site). The highly flexible arachidonic acid binding site is delimited by Phe177, Tyr181, Gln363, His367, Leu368, His372, Ile406, Ala410, Leu414, Leu420, Phe421, Asn425, Pro529, His600, Ala603, Ala606, and Leu607 [102]. Due to the structural changes required to make the binding pocket accessible, these residues were kept as “flexible” in the Induced Fit molecular docking experiments and were used to evaluate the binding of the secondary metabolites to the target protein.

**Figure 6:**
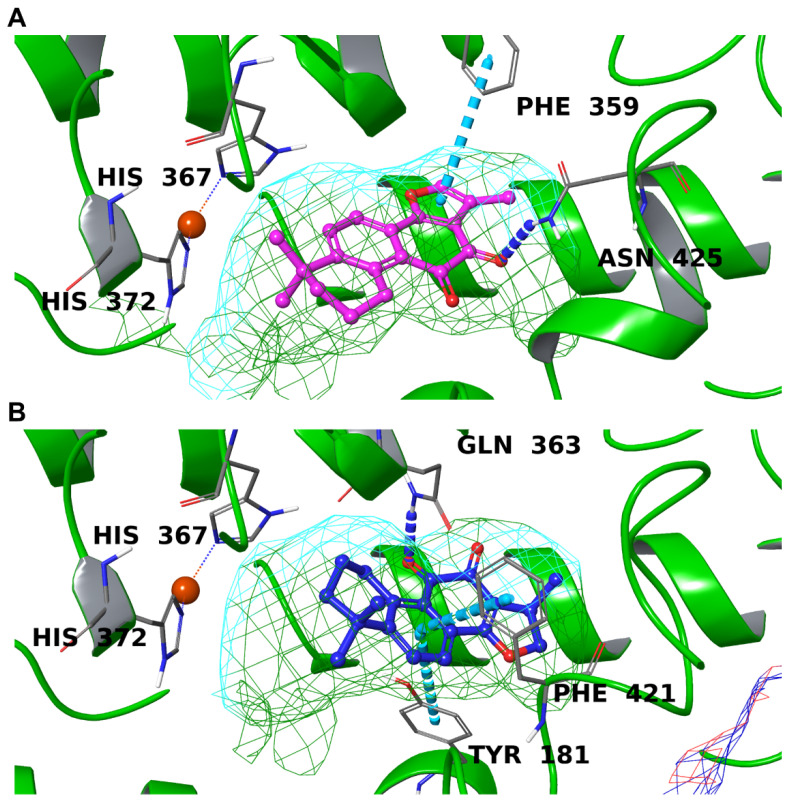
Binding mode of TIIA (**A**) and CRY (**B**) inside the binding pockets of 5-LO. Hydrogen bonds are depicted as blue dotted lines, and π-π stackings are represented by cyan dotted lines. The molecular surface of the binding site is shown in mesh mode and colored according to residue property: green for hydrophobic residues and cyan for polar ones. The important residues of the binding site are labeled in black, and the iron is represented by an orange sphere.

Like for COX-2, TIIA was arranged differently with respect to CRY in the 5-LO binding pocket, with the carbonyl moiety facing a hydrophobic surface (Figure 6A,B). Though one of the carbonyl oxygens formed a hydrogen bond with Asn425, an important residue for inhibitor binding [102], this is not sufficient to match the optimal binding mode of CRY, probably due to the higher flexibility of this compound that can adapt to the cavity better than TIIA. In detail, the latter molecule orientated its polar moiety towards the hydrophilic part of the binding site, forming a hydrogen bond with Gln363. Moreover, two additional π-π stacking interactions were made with Tyr181 and Phe421, both key residues of the 5-LO binding site. As none of the ligands showed an interaction with the iron, it can be assumed that TIIA and, more importantly, CRY represent “canonical” competitive inhibitors of 5-LO.

#### 3.1.4. mPGES-1

**Figure 7:**
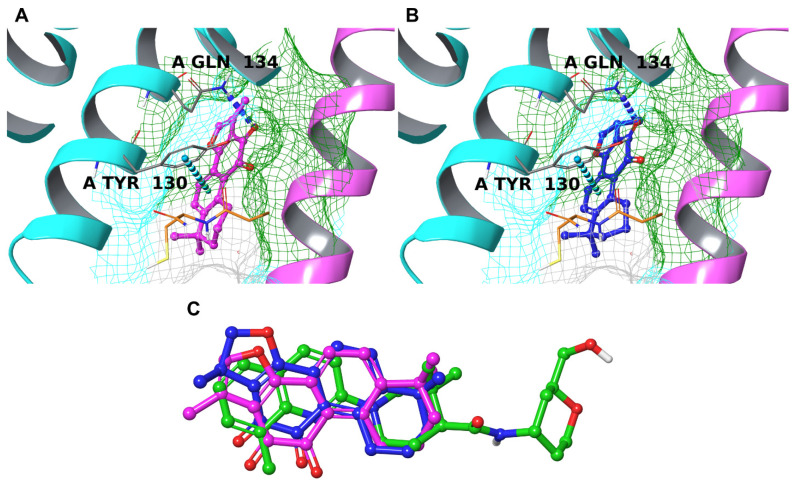
Binding mode of TIIA (**A**) and CRY (**B**) inside the binding pockets of mPGES-1 (cyan ribbons for chain A and pink ribbons for chain B). Hydrogen bonds are depicted as blue dotted lines and π-π stackings are represented by cyan dotted lines. The molecular surface of the binding site is shown in mesh mode and colored according to residue property: green for hydrophobic residues and cyan for polar ones. The interacting residues are labeled in black, and the glutathione molecule is represented by thin tubes (orange carbons). Superposition of TIIA (pink carbons), CRY (blue carbons), and the co-crystallized ligand of mPGES-1 (green carbons) (**C**).

mPGES-1 is a homotrimer that, therefore, contains three distinct binding sites located at the interface between two chains and contains a GSH molecule as a co-substrate in each cavity. As reported [19,65,90,91,103], the binding site is delimited by Arg70, Arg110, Arg126, Ser127, Tyr130, Thr131, and Gln134 on chain A and Tyr28, Ile32, Arg38, Phe44, Asp49, and His53 on chain B. The cavity is small, and it is partially occupied by the GSH molecule, which interacts with a head group of the substrate PGH_2_, causing the lipophilic tails to accommodate outside the binding site [19]. From the visual inspection of the binding poses, the two compounds established interesting interactions with Tyr130_chainA_ and Gln134_chainA_, preventing access to GSH (Figure 7A,B), and were arranged like the co-crystallized inhibitor (Figure 7C). Interestingly, CRY, due to its chemical structure, is able to orientate its methyl moiety towards a hydrophobic cavity formed on chain B (pink ribbons in Figure 7A,B), improving the overall quality of the binding with the counterpart.

#### 3.1.5. PAFR

**Figure 8:**
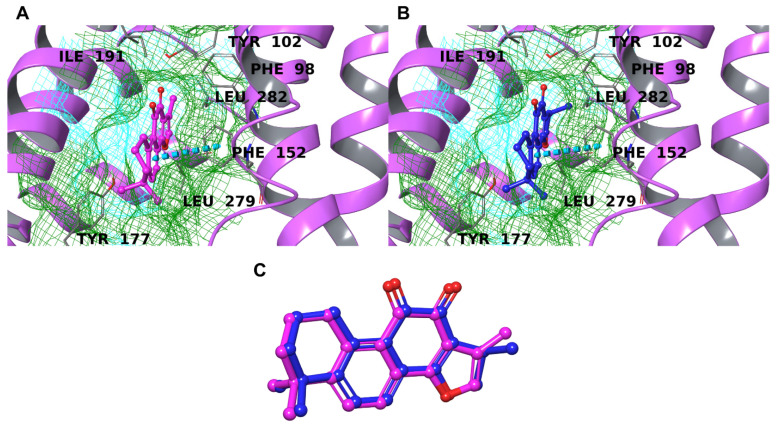
Binding mode of TIIA (**A**) and CRY (**B**) inside the binding pocket of PAFR (lilac ribbons). The π-π stackings are represented by cyan dotted lines. The molecular surface of the binding site is shown in mesh mode and colored according to residue property: green for hydrophobic residues and cyan for polar ones. The important residues of the binding pocket are labeled in black. (**C**) Superimposition of TIIA (magenta) and CRY (blue).

The PAFR is a G-protein-coupled receptor that is expressed on several cell types of membranes and is activated by the platelet-activating factor (PAF) [104]. Like the other members of its family, the PAFR contains 7 transmembrane helices with the binding site located in the extracellular N-terminal domain. The major interactions are hydrophobic due to the lipophilic nature of the cavity; in particular, important amino acids are Tyr22, Trp73, Tyr77, Phe97, Trp255, His275, and Leu279 (Table 1) [47]. The spatial arrangement shown by the two compounds was very similar (Figure 8C), with an almost complete overlap between TIIA and CRY. As expected, there is a unique non-hydrophobic (π-π stacking) interaction with Phe152, while the rest of the energetic contribution was represented by lipophilic contacts with the extended non-polar surface (Figure 8A,B). The predicted binding energies were slightly better than the one calculated for the co-crystallized ligand, despite the latter making a higher number of interactions with the target because it is bigger and with more “interacting points” with respect to the small natural compounds. Overall, considering either the predicted binding energy and the mode of interaction, both TIIA and CRY seemed to be promising ligands for this protein.

#### 3.1.6. EP3 and EP4

These two G-protein-coupled receptors interact with PGE_2_ and modulate some biological activities according to the tissue they are expressed in. In detail, EP3 is involved in fever generation, angiogenesis, smooth muscle contraction, and, obviously, thrombosis [48]; EP4, on the other hand, is also expressed on lymphocytes and seems to trigger the conversion of Th1 into Th17 cells [49,105].

**Figure 9:**
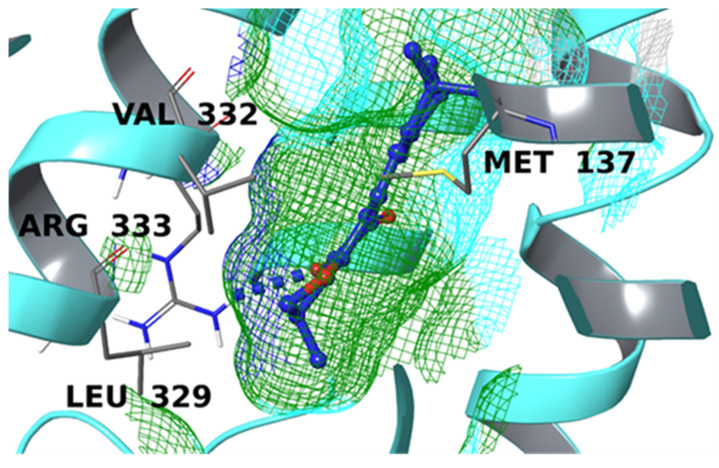
Binding mode CRY inside the binding pocket of EP3 (cyan ribbons). The hydrogen bond is represented by blue dotted lines. The molecular surface of the binding site is shown in mesh mode and colored according to residue property: green for hydrophobic residues, blue for positively charged amino acids, and cyan for polar ones. The important residues of the binding pocket are labeled in black.

The EP3 binding site contains three important residues (Thr206, Arg333, and Tyr114), which stabilize the carboxyl group of PGE_2_; the alkyl chains, instead, are accommodated on the hydrophobic surface of the cavity, reaching the receptor surface, and interact with Met137, Phe209, and Val332. Additional interactions are made with Met58 and Asp99. In this case, only CRY showed a satisfying binding mode, forming a hydrogen bond with Arg333 and interacting in a non-polar manner with Met138, Val332, and Leu329 (Figure 9). The molecule accommodated well in the pocket, directing the oxygens toward the positively charged surface and the remaining lipophilic moiety towards the hydrophobic surface.

**Figure 10:**
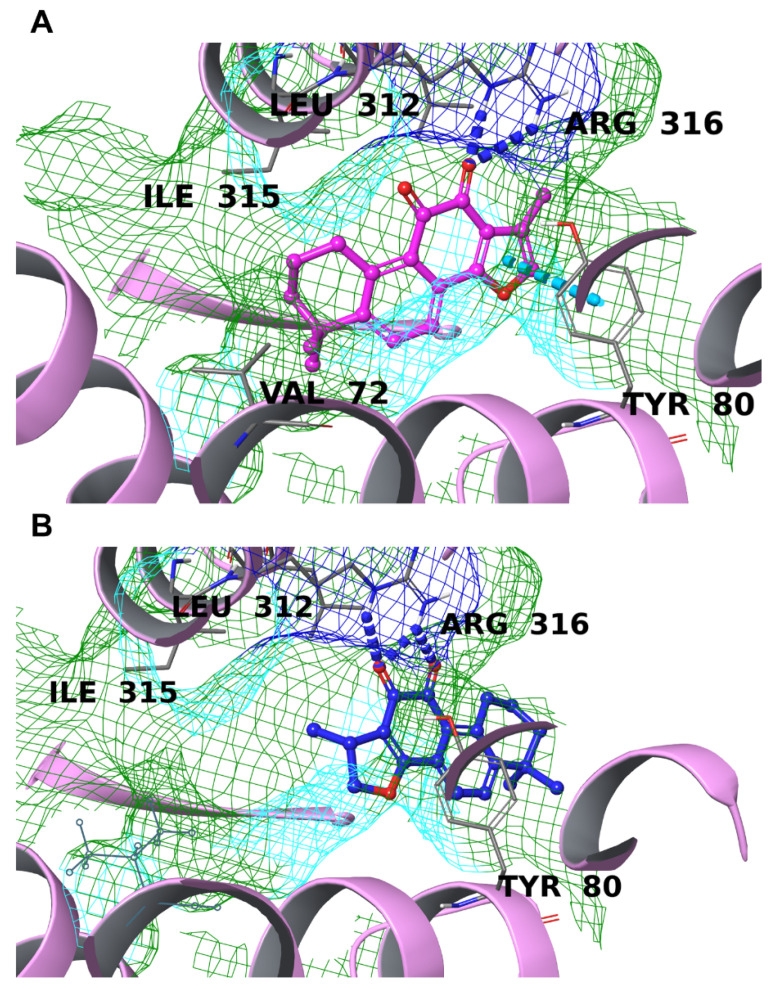
Binding mode TIIA (**A**) and CRY (**B**) inside the binding pocket of EP4 (light pink ribbons). The hydrogen bonds are represented by blue dotted lines, and the π-π stacking is represented by a cyan dotted line. The molecular surface of the binding site is shown in mesh mode and colored according to residue property: green for hydrophobic residues, blue for positively charged residues, and cyan for polar ones. The important residues of the binding pocket are labeled in black.

The EP4 binding cavity is very similar to that of EP3 for both its extended hydrophobic surface and the significant role played by Arg316, which coordinates the carboxyl moiety of PGE_2_ like EP3. Other significant residues are Thr168, Trp169, and Leu312, highly conserved in the GPCR family, and Val72, Leu99, Ile315, Ser319, and Val320, which are specific for EP4 [49]. TIIA interacted successfully with the counterpart, making a double hydrogen bond with Arg316 and a π-π interaction with Tyr80; the three-dimensional disposition was also favorable, with the carbonyl oxygens pointing at the positively charged surface and the rest of the molecule accommodated on the hydrophobic part of the cavity. Conversely, CRY was not well-accommodated in this receptor, with multiple hydrogen bonds with Arg316 as the only relevant interaction; moreover, the molecule was located far from the optimal position, almost outside the binding pocket (Figure 10).

#### 3.1.7. MMP-1 and MMP-2

**Figure 11:**
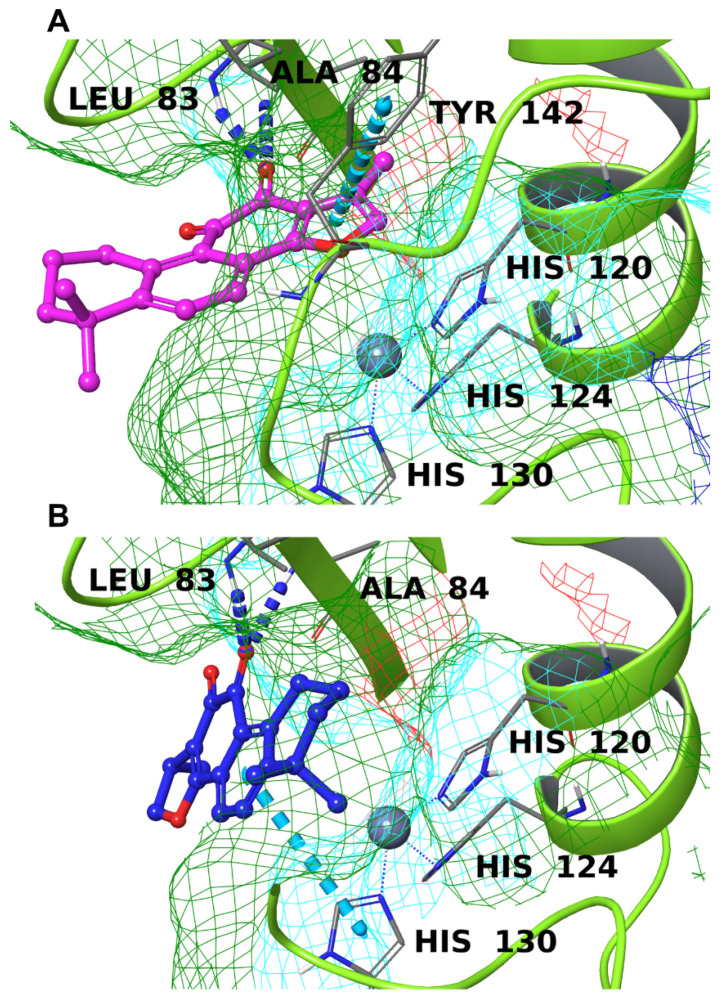
Binding mode TIIA (**A**) and CRY (**B**) inside the binding pocket of MMP-2 (lime green ribbons). The hydrogen bonds are represented by blue dotted lines, and the π-π stackings are represented by cyan dotted lines. The molecular surface of the binding site is shown in mesh mode and colored according to residue property: green for hydrophobic residues, blue for positively charged residues, and cyan for polar ones. The important residues of the binding pocket are labeled in black, and the zinc atom is depicted as a gray sphere.

From the data reported in Figure 3, it was clear that neither TIIA nor CRY showed an adequate binding to MMP-1 due to the high polarity of the binding surface, which prevented a good accommodation of these lipophilic compounds inside the cavity (data not shown). Concerning MMP-2, the data were slightly better, though not satisfactory. Both molecules made two hydrogen bonds with Leu83 and Ala84, through one of the carbonyl oxygens, and a π-π stacking with Tyr142 and His130, respectively, for TIIA and CRY (Figure 11). Despite the interactions with relevant amino acids, the spatial arrangement these two compounds showed was not optimal: the considerable polarity of the pocket prevented the good insertion of TIIA and CRY, leaving them in the shallow part of the binding site. 

#### 3.1.8. Summing Up

From the in-silico data obtained with the molecular docking experiments, both TIIA and CRY showed a discrete interaction with the considered target. In detail, COX-2, 5-LO, PAFR, and mPGES-1 appeared as the most likely protein counterpart for these natural compounds, considering the binding mode and the number of interactions with key binding site residues. For this reason, they were selected for further in vitro and in vivo studies.

### 3.2. In Vitro Experiments

#### Effect of TIIA and CRY on mPGES-1, 5-LO, COX-1, and COX-2 in Cell-Free Assays

The cell-free assay was used to assess the inhibition of mPGES-1 by TIIA and CRY. To do so, the human mPGES-1 in the microsomal fraction of IL-1β-stimulated A549 cells was assayed. The two compounds were added at a concentration of 10 µM to the microsomal fraction, and 20 µM PGH_2_ as substrate was added to determine the inhibitory activity of the enzyme. From the data collected, it emerged that CRY had a discrete inhibitory activity against mPGES-1 with an IC_50_ = 1.9 ± 0.4 µM, while TIIA showed no relevant interference with the target. These data are consistent with the predicted binding affinity of CRY to mPGES-1 and a number of interactions higher than the counterpart TIIA.

**Figure 12:**
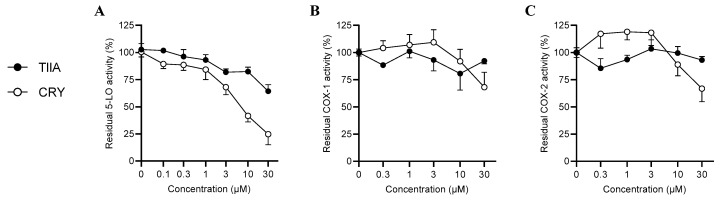
Effect of TIIA and CRY on the activity of 5-LO (**A**), COX-1 (**B**), and COX-2 (**C**). Data are presented as means ± SEM of residual enzyme activity after incubation of isolated 5-LO (*n* = 7), COX-1 (*n* = 3), and COX-2 (*n* = 4) with TIIA or CRY at the indicated concentrations. Statistical analysis was conducted by non-constrained non-linear regression of inhibitor vs. response (variable slope).

Cell-free assays using isolated enzymes were employed to assess the inhibition of 5-LO, COX-1, and COX-2 by TIIA and CRY. Thus, the isolated enzymes were incubated with TIIA or CRY (0.3 to 30 µM), and product formation was assessed by HPLC-UV. Compared to vehicle-treated controls, CRY exhibited a discrete inhibitory activity against 5-LO with an IC_50_ of 7.1 µM (Figure 12). CRY also inhibited COX-1 and COX-2 activity, albeit to a minor degree (IC_50_ > 30 µM) compared to observations with 5-LO. As shown in Table 2, the overall inhibition of the tested enzymes was from 1.3 to 2.6-fold higher for CRY than for TIIA. These data support the predicted higher affinity of CRY compared to its counterpart TIIA.

### 3.3. Effect of TIIA and CRY on EP Receptor

**Figure 13:**
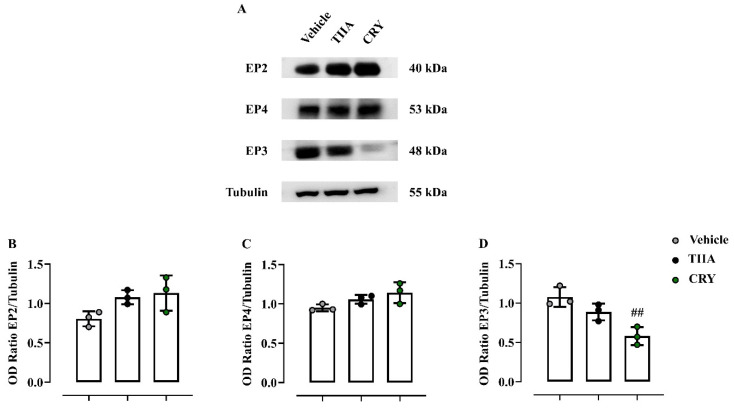
Effect of TIIA and CRY on EPs receptor. Representative western blotting (**A**) and related cumulative densitometric analyses of EP2 (**B**), EP4 (**C**), EP3 (**D**) of clot homogenates from mice injected i.p. with TIIA (10 mg/kg) or CRY (10 mg/kg) 1 h before the experiments. Data are presented as means ± SD of three separate independent experiments run each with *n* = 7 mice per group pooled. Statistical analysis was conducted by using one-way ANOVA followed by Bonferroni’s for multiple comparisons. ^##^ *p* < 0.01 vs. vehicle group.

Since PGE_2_ has been reported to exhibit a biphasic effect on platelet aggregation via EP receptors, we sought to examine on clot homogenates, the effect of TIIA and CRY on EP2, EP3, and EP4 by western blot analysis. Representative results presented in Figure 13A show that both diterpenoids did not alter the expression of EP2 (Figure 13B) and EP4 (Figure 13C). Interestingly, a significant reduction of EP3 expression in CRY (but not TIIA)-treated mice compared to the vehicle group (*p* ≤ 0.01) was found (Figure 13D). Densitometric values are expressed as an OD ratio against tubulin. The uncropped and triplicate of the original Western blots are presented in Supplementary Figures S1–S4.

### 3.4. Effect of TIIA and CRY on Clot Retraction and Bleeding Time

**Figure 14:**
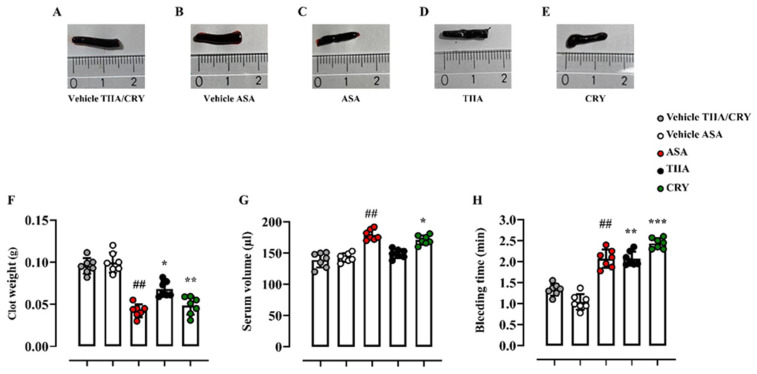
Effect of TIIA and CRY on in vivo clot retraction and bleeding time. Not-anticoagulated blood samples were incubated into Microvette^®^ 300 Z, containing clotting activator, at room temperature for 2 h. The impact of TIIA and CRY on platelet activation was evaluated by clot morphology (**A**–**E**), quantification of clot weights (**F**), and residual serum volumes (**G**). The effect on platelet hemostatic function and thrombotic activity was also determined by tail bleeding time (**H**). TIIA (10 mg/kg), CRY (10 mg/kg), or ASA (10 mg/kg) were administered i.p. to CD-1 mice 1 h before the experiments. Data are presented as means ± SD of *n* = 7 mice per group. Statistical analysis was conducted by one-way ANOVA followed by Bonferroni’s for multiple comparisons. ^##^ *p* ≤ 0.01 vs. vehicle ASA; * *p* ≤ 0.05, ** *p* ≤ 0.01, *** *p* ≤ 0.001 vs. vehicle TIIA/CRY.

The effect of TIIA and CRY was investigated on platelet activation by performing an ex vivo model of clot retraction. Results from this in vivo model were assessed macroscopically by the evaluation of clot morphology (Figure 14A–E) and numerically (clot score) by clot weights and residual serum volumes. It was observed that samples from TIIA- and CRY-treated mice (10 mg/kg; i.p.) were much less retracted (TIIA: 0.068 ± 0.009 g, *p* ≤ 0.05; CRY: 0.048 ± 0.011 g, *p* ≤ 0.01) compared to the vehicle group (0.096 ± 0.009 g), suggesting that both diterpenoids significantly decreased the clot retraction rates of platelets. A similar effect was found for ASA-treated mice (0.042 ± 0.008 g vs. 0.099 ± 0.012 g, *p* ≤ 0.01) (Figure 14F). Congruently, as shown in Figure 14G, the CRY-treated group resulted in a more prominent production of serum compared to the vehicle group (171.0 ± 7.35 μL vs. 139 ± 12.08 μL, *p* ≤ 0.05). Successively, in order to support the hypothesis that both diterpenoids could affect hemostasis and thrombus formation, in vivo tail bleeding assay was performed [106]. As shown in Figure 14H, pretreatment with TIIA and CRY significantly increased the bleeding time in mice (TIIA: 2.07 ± 0.17 min, *p* ≤ 0.01; CRY: 2.44 ± 0.12 min, *p* ≤ 0.001) compared to the vehicle group (1.33 ± 0.14 min). Similar results were observed after ASA administration (2.07 ± 0.22 min vs. 1.04 ± 0.19 min, *p* ≤ 0.01).

## 4. Discussion

Inflammation and hemostasis cannot be considered as two separate processes since there are several connecting points making them part of a unique, defensive host response. There is much evidence that inflammation triggers hemostatic imbalance. Experimental and clinical data demonstrate that inflammation is associated with an increased risk of cardiovascular events [107,108,109]. An important aspect to be considered when the link between hemostasis and inflammation is examined is the contribution of platelets to both processes [110,111]. Platelets are considered effective elements of the inflammatory system. Under physiological conditions, platelets circulate freely in the blood. On the contrary, when the endothelium is damaged, platelets adhere to collagen fibers of the sub-endothelium, thereby becoming activated. Activated platelets express molecules on their surface driving platelet endothelium adhesion and platelet–leukocytes interaction. In this work, a combined in silico, in vitro, in vivo, and ex-vivo approach was performed to prove a possible use of tanshinones (TIIA and CRY) in the prevention of thrombotic events. Our attention was focused on ten key targets of the inflammatory/pro-thrombotic cascade: sPLA_2_, COX-1, COX-2, 5-LO, mPGES-1, EP3, and EP4 receptors, PAFR, MMP-1, and MMP-2. 

From the analysis of the docking poses, it was clear that both compounds are able to interact significantly with COX-2, 5-LO, PAFR, and mPGES-1, which are key proteins involved not only in the onset of inflammation but also with a cardiovascular alteration of platelet activity (and hyper-reactivity) and, to a lesser extent, thrombotic and thromboembolic disease [112,113]. Moreover, a difference in the binding to the two isoforms of COX was highlighted, suggesting a preferential binding of CRY to COX-2. This compound seemed to adapt better to the binding pocket of several of the considered enzymes due to its less rigid chemical structure. This may suggest that CRY acts as a multitarget rather than a selective inhibitor. Consistently, results from cell-free COX-1 and COX-2 activity assays revealed the similar moderate inhibition of the two COX isoforms by CRY, while TIIA was essentially inactive up to 30 µM (highest concentration tested). In addition, in 5-LO activity assays using the isolated human recombinant enzyme, CRY displayed more efficient inhibition than TIIA. These data confirmed the hypothesis that CRY is capable of a multitarget inhibitory effect, and its mechanism of action should not be strictly compared to known selective inhibitors (like rofecoxib for COX-2). This broad spectrum of action could represent a significant advantage as it allows for the interference with more than one key player in the thrombotic events, generating a synergism that exerts the biological effect of a low therapeutic dose.

Interestingly, concerning EP3 and EP4, two of the prostaglandin receptors, specular results emerged, with CRY showing a better binding on EP3 and TIIA on EP4. It is important to notice that only with PAFR did the two compounds show an interaction with the target better than the co-crystallized molecule, putting it in a pivotal role as the focus for future work.

Owing to the undesirable effects of COX-2-selective inhibitors (thrombotic events, hypertension, and heart failure), interest was focused on mPGES-1 as an alternative target for the development of analgesics and anti-inflammatory drugs [12]. The thought was that the analgesic efficacy would be largely, if not totally, conserved by PGE_2_ suppression, while most of the cardiovascular risk would be minimized by conserving or even boosting the cardioprotective PGI_2_ production [114]. Indeed, global or myeloid-specific deletion of mPGES-1 has proven efficacy in restraining atherogenesis, attenuating the proliferative response to vascular injury, and limiting aortic aneurysm formation [115,116,117,118,119].

To further support this evidence, an in vivo and ex vivo model of platelet aggregation and blood stasis, namely mouse clot retraction and bleeding time, was also performed. Platelet adhesion to the extracellular matrix in flowing blood is the first and crucial step for thrombus formation. During the platelet adhesion, pseudopodia are formed, which supports an effective attachment to the injured surface of the blood vessel wall [120]. Clot retraction, the shrinking of a blood clot that involves platelet adhesion and aggregation, is a process driven by extracellular signaling that results in the contraction of the fibrin mesh. The ability of platelets to drive clot retraction is a surrogate measure of outside-in signaling [73]. Our results showed that both diterpenoids, and in particular CRY, were able to increase the bleeding time in mice with an effect similar to those observed after ASA administration. Interestingly, results from ex vivo experiments displayed a promising anti-coagulant property of CRY with a profile greater than ASA. 

PGE_2_ acts on four specific GPCR subtypes, termed EP1–4. The activation of these receptors by PGE_2_ or artificial compounds stimulates distinct signal transduction pathways and mediates various biological functions [28]. The EP1 receptor couples to Gq-proteins to increase intracellular Ca^2+^ concentration. The EP2 and EP4 receptors couple to Gs-proteins and evoke an increase in intracellular cAMP concentration. EP3 receptor mainly couples to Gi-proteins to decrease cAMP production, while it has at least eight variants that may activate other different signaling pathways, for example, elevate intracellular Ca^2+^ or activate the small G-protein Rho. The involvement of the four PGE_2_ receptors in cardiovascular function has been studied with various genetic deletion approaches or small molecule agonists or antagonists, and the conclusions varied [10]. Thrombosis is the most pronounced risk signal associated with COX-2 selective NSAIDs [121]. The inhibition of COX-2 or the deletion of IP (PGI_2_ receptor) significantly accelerated thrombogenesis, reflected by the shortened time to vascular occlusion after photochemical injury of the carotid artery and reduced thrombogenesis after laser-induced cremaster arterioles injury; however, these effects were not observed in either global or myeloid cell mPGES-1 knockout mice [115,118]. Effects on both augmented PGI_2_ and suppressed PGE_2_ might be relevant to this beneficial phenotype: PGI_2_ restrains thrombogenesis, while PGE_2_ elicits platelet aggregation at low concentrations via EP3 [29]. PGE_2_ has been reported to exhibit a biphasic effect on platelet aggregation. While EP1 expression is lacking, the other three PGE_2_ receptors, EP2, EP3, and EP4, are all expressed in platelets, and the expression level of EP3 is much higher than EP2 and EP4. In detail, EP3 mediates the pro-aggregatory effect of PGE_2_. EP3 agonists had shown concentration-dependent potentiation of platelet aggregation in vitro [29]. In vivo, EP3 gene depletion mice showed significantly prolonged tail bleeding time and when challenged with arachidonic acid, lung thrombus formation, and mortality [30]. In addition, by mechanical rupture of the plaque with scratching in a murine model, the atherothrombosis was drastically decreased when there was a lack of EP3 in platelets [122]. Indeed, DG-041, a direct-acting EP3 antagonist, has been considered as an effective antiplatelet and anti-atherothrombosis drug without increasing the bleeding risk [31,123]. In contrast, the EP2 and EP4 signaling mediate the anti-aggregatory effects of PGE_2_, albeit the IP plays the predominant inhibitory role at higher PGE_2_ concentrations. Notably, these inhibitory effects of EP2 and EP4 might only be efficient when the EP3 receptor is absent [124]. Nevertheless, PGE_2_ sensitizes platelets to their agonists, such as thrombin or collagen through the activation of its EP3 receptor, while PGE_2_ inhibits platelet activity through EP2 and EP4 receptors [124]. Notably, our results shown that CRY, but not TIIA, was able to reduce the expression of EP3 isoform on mice clots without interfering with EP2 and EP4 enzymatic activity. This is of extreme importance from the perspective of a selective blockade of the EP3 activity (and/or activation of EP2 or EP4) in view of rational strategies for developing novel antiplatelet agents and/or preventing thrombogenesis. All these findings are strengthened by the lack of toxic effects and by a low cytotoxic profile for tanshinones in both human and murine cell lines [125,126,127]. Moreover, recent reports have revealed that both TIIA and CRY are able to selectively induce apoptosis in different cancer cell lines in a concentration and time-dependent manner [128,129,130].

## 5. Conclusions

In conclusion, this study aimed to use a translational approach to investigate and rationalize the anti-aggregating properties of two secondary metabolites of *S. miltiorrhiza* Bunge. First, molecular docking experiments were carried out on key targets of the inflammatory/thrombogenic pathway to highlight putative partners. The binding poses were evaluated in terms of energy and interactions with the key residues of the protein counterparts. From the analysis of the corresponding results, COX-2, 5-LO, PAFR, and mPGES-1 emerged as the most probable interacting macromolecules for the two compounds. After the rationalization at the molecular level of the ligand-protein complexes, with in vitro, in vivo, and ex-vivo studies, the predicted binding was confirmed, highlighting a preferential multitarget action of CRY. The results showed, for the first time, that TIIA, and in particular CRY, are able to interact significantly with the key proteins involved not only in the onset of inflammation but also in platelet activity (and hyper-reactivity). Future preclinical and clinical investigations, together with this evidence, could provide the scientific basis to consider these compounds as an alternative therapeutic approach for thrombotic- and thromboembolic-based diseases.

## Figures and Tables

**Table 1:** Important binding site residues for each target. For mPGES-1, the different chains are reported in brackets.

Protein	Binding Site Residues
sPLA_2_ [87,88]	Phe5, His6, Arg7, Lys10, Gly29, His47, Asp48, Lys61, Asp89, Phe93, Phe98, His115, Arg117, and Arg122
COX-1 [84,85]	His90, Leu93, Val116, Arg120, Gln192, Ala201, Phe205, Phe209, Val228, Tyr 248, Val344, Tyr348, Val349, Leu352, Ser353, Tyr355, Leu359, Ile377, Phe381, Leu384, Tyr385, Trp387, His513, Ile517, Phe518, Met522, Ile523, Glu524, Gly526, Ala527, Ser530, and Leu534
COX-2 [84,85]	His90, Leu93, Val116, Arg120, Gln192, Ala201, Phe205, Phe209, Val228, Tyr 248, Val344, Tyr348, Val349, Leu352, Ser353, Tyr355, Leu359, Ile377, Phe381, Leu384, Tyr385, Trp387, Arg513, Ile517, Phe518, Met522, Val523, Glu524, Gly526, Ala527, Ser530, and Leu534
5-LO [82,89,90]	Phe177, Tyr181, Gln363, Leu368, Ile406, Lys409, Arg411, Leu414, Leu420, Phe421, Asn425, Trp599, and Leu607
mPGES-1 [82,86,91]	Arg70(A), Arg110(A), Arg126(A), Ser127(A), Tyr130(A), Thr131(A), Gln134(A), Tyr28(B), Ile32(B), Arg38(B), Leu39(B), Phe44(B), Asp49(B), and His53(B)
PAFR [47]	Tyr22, Trp73, Tyr77, Phe97, Phe98, Thr101, Tyr102, Phe152, Glu175, Tyr177, His188, Ile191, His248, Gln252, Trp255, His275, Leu279, and Leu282
EP3 [48]	Pro55, Met58, Asp99, Gln103, Thr106, Thr107, Val110, Tyr114, Met137, Thr206, Trp207, Phe209, Trp295, Leu298, Leu329, Val332, Arg333, Ser336, and Gln339,
EP4 [49]	Pro24, Val72, Thr76, Tyr80, Leu99, Thr168, Trp169, Leu312, Ile315, Arg316, Ser319, and Val320
MMP-1 [50,83]	Arg114, Val115, His118, Glu119, Leu135, Tyr137, Phe138, Ser139, Tyr140, Asn180, Leu181, Ala182, His183, and Glu219
MMP-2 [51]	Leu82, Leu83, Ala84, His85, Leu116, Val117, His120, Leu137, Ala139, Pro140, Ile141, Tyr142, Thr143, Thr145, and Leu150

**Table 2:** Residual activity of 5-LO, COX-1, and COX-2 after incubation with TIIA or CRY at 30 µM.

Compound	Residual Activity (% of Vehicle-Treated Enzymes)		
	5-LO	COX-1	COX-2
TIIA	64.3	92.4	93.1
CRY	24.7	68.2	66.8

## Data Availability

Not applicable.

## Supplementary Materials

The following supporting information can be downloaded at: https://www.mdpi.com/article/10.3390/biom12010099/s1. Original blot images (Figures S1–S4), redocking binding poses with corresponding RMSD values (Figure S5), and Ballesteros–Weinstein numbering scheme of PAFR, EP3, and EP4.

## Abbreviations

12-HHT: 12-hydroxyheptadecatrienoic acid; 5-LO, 5-lipoxygenase; ASA, acetylsalicylic acid; COX-, cyclooxygenase-; cPGES, cytosolic prostaglandin E_2_ synthase; CRY, Cryptotanshinone; EGF, epidermal growth factor; EP, endothelial prostaglandin; ERKs, extracellular signal-regulated kinases; GPCR, G-protein-coupled receptors; GSH, glutathione; i.p., intraperitoneally; IL-, interleukin-; LTs, leukotrienes; MMPs, matrix metalloproteinase; mPGES-, microsomal prostaglandin E synthase-; NSAIDs, non-steroidal anti-inflammatory drugs; PAF, platelet-activating factor; PAFR, platelet-activating factor receptor; PBS-T, PBS-Tween 20; PGs, prostaglandins; sPLA_2_, secreted phospholipase A_2_; RT, room temperature; TIIA, Tanshinone IIA; TXA, thromboxane.
