# Supplementary material for: In Silico, In Vitro and In Vivo Analysis of Tanshinone IIA and Cryptotanshinone from Salvia miltiorrhiza as Modulators of Cyclooxygenase-2/mPGES-1/Endothelial Prostaglandin EP3 Pathway
Source: Biomolecules. 2022 Jan 7;12(1):99. doi: 10.3390/biom12010099 (PMC8774285; doi:10.3390/biom12010099)
Supplement: Supplementary file 1 [file biomolecules-12-00099-s001.zip › biomolecules-1517096-supplementary.pdf]

Supplementary Figures

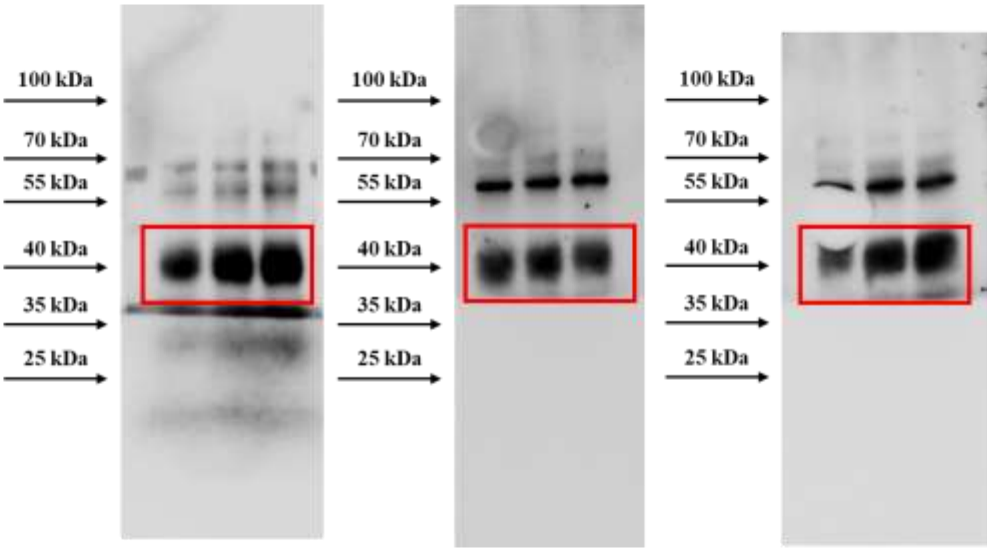

Figure S1. Original western blots for EP2 obtained from clot homogenates in all experimental conditions run each with n = 7 mice per group pooled.

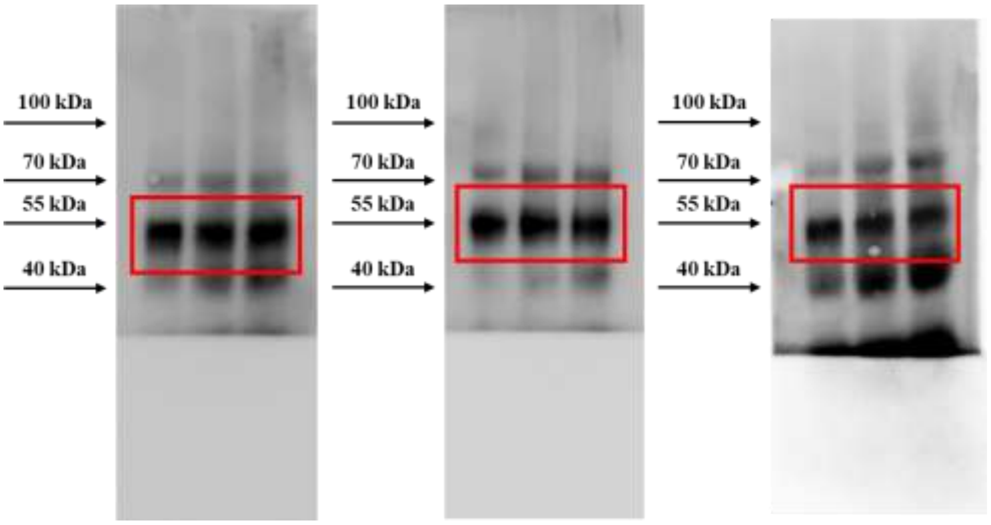

Figure S2. Original western blots for EP4 obtained from clot homogenates in all experimental conditions run each with n = 7 mice per group pooled.

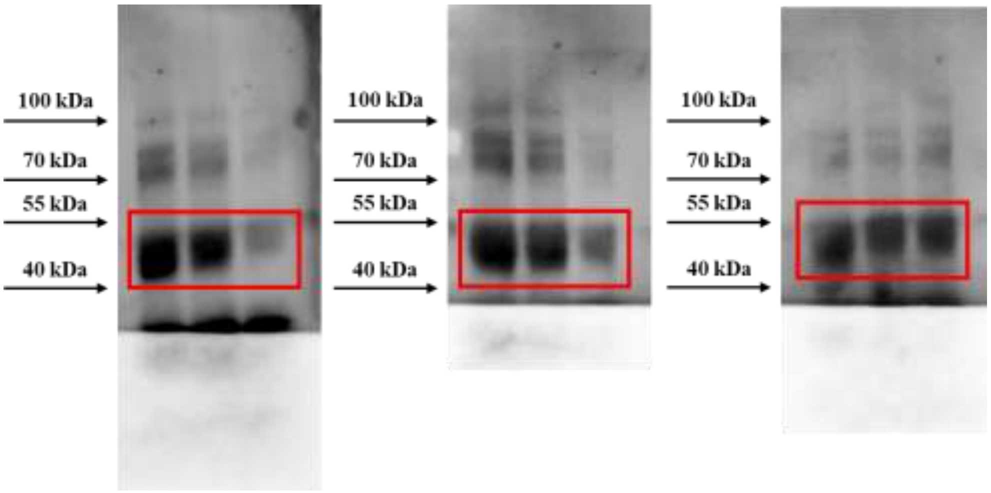

Figure S3. Original western blots for EP3 obtained from clot homogenates in all experimental conditions run each with n = 7 mice per group pooled.

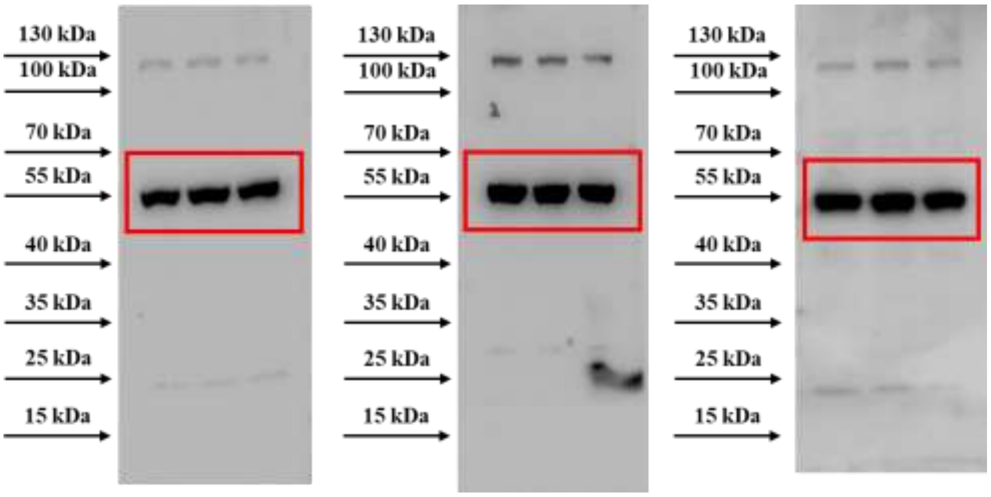

Figure S4. Original western blots for tubulin obtained from clot homogenates in all experimental conditions run each with n = 7 mice per group pooled.

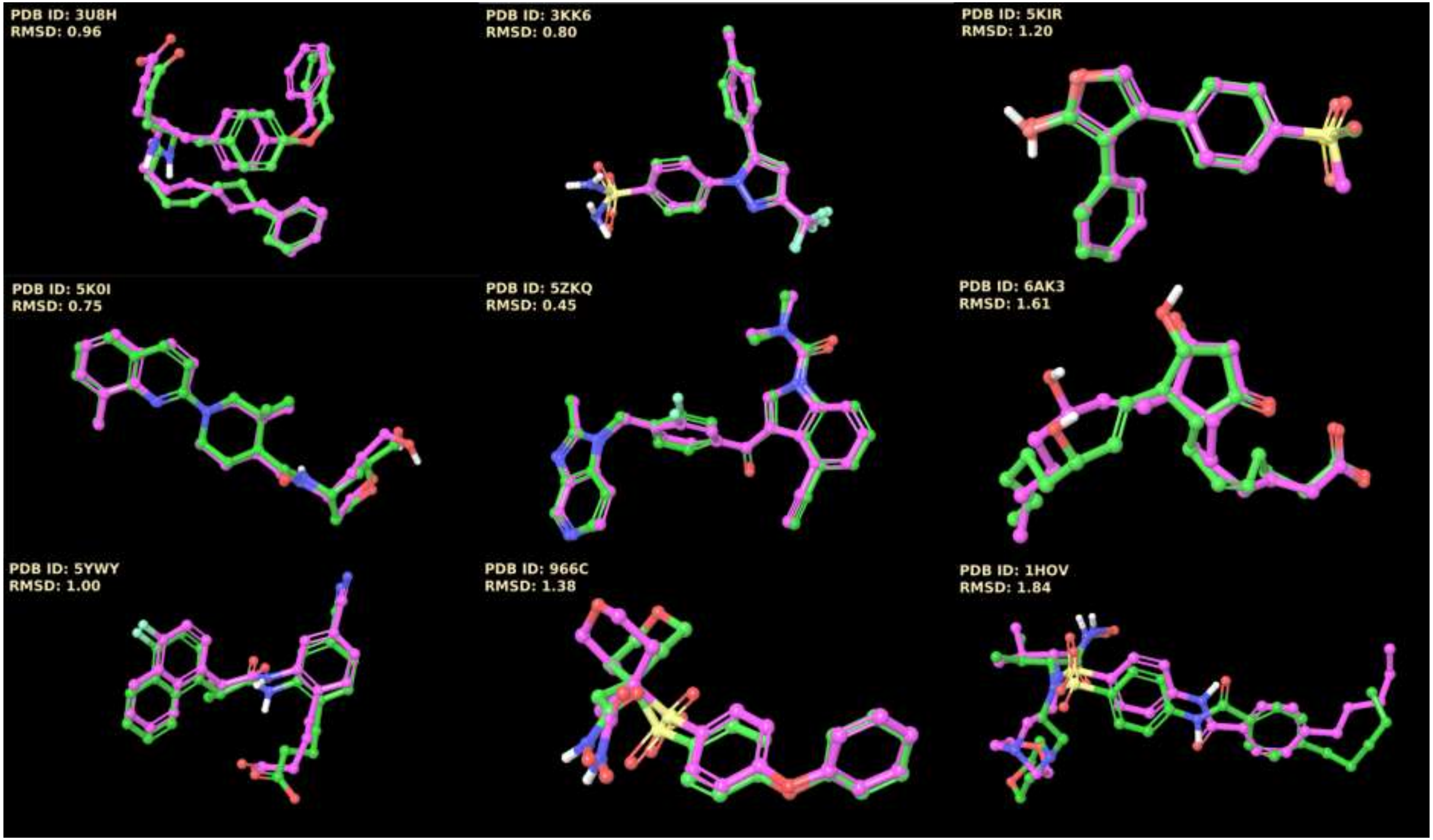

Figure S5. RMSD values of the redocking experiments of $sPLA_2$ (3UH8), COX-1 (3KK6), COX-2 (5KIR), mPGES-1 (5K0I), PAFR (5ZKQ), EP3 (6AK3), EP4 (5YWY), MMP1 (996C), and MMP2 (1HOV).

Ballesteros–Weinstein numbering scheme

PAFR

Residue Number	B-W Numbering
14	1.31
15	1.32
16	1.33
17	1.34
18	1.35
19	1.36
20	1.37
21	1.38
22	1.39
23	1.4
24	1.41
25	1.42
26	1.43
27	1.44
28	1.45
29	1.46
30	1.47
31	1.48
32	1.49
33	1.5
34	1.51
35	1.52
36	1.53
37	1.54
38	1.55
39	1.56
40	1.57
41	1.58
42	1.59
54	2.42
55	2.43
56	2.44
57	2.45
58	2.46
59	2.47
60	2.48
61	2.49
62	2.5
63	2.51
64	2.52
65	2.53
66	2.54
67	2.55
68	2.56
69	2.57
70	2.58
71	2.59
72	2.6
73	2.61
74	2.62
75	2.63
76	2.64
77	2.65
78	2.66
79	2.67
86	3.21
87	3.22
88	3.23
89	3.24
90	3.25
91	3.26
92	3.27
93	3.28
94	3.29
95	3.3
96	3.31
97	3.32
98	3.33
99	3.34
100	3.35
101	3.36
102	3.37
103	3.38
104	3.39
105	3.4
106	3.41
107	3.42
108	3.43
109	3.44
110	3.45
111	3.46
112	3.47
113	3.48
114	3.49
115	3.5
116	3.51
117	3.52
118	3.53
119	3.54
131	4.39
132	4.4
133	4.41
134	4.42
135	4.43
136	4.44
137	4.45
138	4.46
139	4.47
140	4.48
141	4.49
142	4.5
143	4.51
144	4.52
145	4.53
146	4.54
147	4.55

148	4.56
149	4.57
183	5.35
184	5.36
185	5.37
186	5.38
187	5.39
188	5.4
189	5.41
190	5.42
191	5.43
192	5.44
193	5.45
194	5.46
195	5.47
196	5.48
197	5.49
198	5.5
199	5.51
200	5.52
201	5.53

202	5.54
203	5.55
204	5.56
205	5.57
206	5.58
207	5.59
208	5.6
227	6.29
228	6.3
229	6.31
230	6.32
231	6.33
232	6.34
233	6.35
234	6.36
235	6.37
236	6.38
237	6.39
238	6.4
239	6.41
240	6.42

241	6.43
242	6.44
243	6.45
244	6.46
245	6.47
246	6.48
247	6.49
248	6.5
249	6.51
250	6.52
251	6.53
252	6.54
253	6.55
254	6.56
255	6.57
256	6.58
257	6.59
258	6.6
276	7.36
277	7.37
278	7.38

279	7.39
280	7.4
281	7.41
282	7.42
283	7.43
284	7.44
285	7.45
286	7.46
287	7.47
288	7.48
289	7.49
290	7.5
291	7.51
292	7.52
293	7.53
294	7.54
295	7.55
296	7.56

EP3

Residue number	B-W Numbering
47	1.31
48	1.32
49	1.33
50	1.34

51	1.35
52	1.36
53	1.37
54	1.38
55	1.39
56	1.4

57	1.41
58	1.42
59	1.43
60	1.44
61	1.45
62	1.46

63	1.47
64	1.48
65	1.49
66	1.5
67	1.51
68	1.52

69	1.53
70	1.54
71	1.55
72	1.56
73	1.57
74	1.58
75	1.59
91	2.42
92	2.43
93	2.44
94	2.45
95	2.46
96	2.47
97	2.48
98	2.49
99	2.5
100	2.51
101	2.52
102	2.53
103	2.54
104	2.55
105	2.56
106	2.57
107	2.58
108	2.59
109	2.6
110	2.61
111	2.62
112	2.63
113	2.64

114	2.65
115	2.66
116	2.67
126	3.21
127	3.22
128	3.23
129	3.24
130	3.25
131	3.26
132	3.27
133	3.28
134	3.29
135	3.3
136	3.31
137	3.32
138	3.33
139	3.34
140	3.35
141	3.36
142	3.37
143	3.38
144	3.39
145	3.4
146	3.41
147	3.42
148	3.43
149	3.44
150	3.45
151	3.46
152	3.47

153	3.48
154	3.49
155	3.5
156	3.51
157	3.52
158	3.53
159	3.54
171	4.39
172	4.4
173	4.41
174	4.42
175	4.43
176	4.44
177	4.45
178	4.46
179	4.47
180	4.48
181	4.49
182	4.5
183	4.51
184	4.52
185	4.53
186	4.54
187	4.55
188	4.56
189	4.57
226	5.35
227	5.36
228	5.37
229	5.38

230	5.39
231	5.4
232	5.41
233	5.42
234	5.43
235	5.44
236	5.45
237	5.46
238	5.47
239	5.48
240	5.49
241	5.5
242	5.51
243	5.52
244	5.53
245	5.54
246	5.55
247	5.56
248	5.57
249	5.58
250	5.59
251	5.6
276	6.29
277	6.3
278	6.31
279	6.32
280	6.33
281	6.34
282	6.35
283	6.36

284	6.37
285	6.38
286	6.39
287	6.4
288	6.41
289	6.42
290	6.43
291	6.44
292	6.45
293	6.46
294	6.47
295	6.48

296	6.49
297	6.5
298	6.51
299	6.52
300	6.53
301	6.54
302	6.55
303	6.56
304	6.57
305	6.58
306	6.59
307	6.6

329	7.36
330	7.37
331	7.38
332	7.39
333	7.4
334	7.41
335	7.42
336	7.43
337	7.44
338	7.45
339	7.46
340	7.47

341	7.48
342	7.49
343	7.5
344	7.51
345	7.52
346	7.53
347	7.54
348	7.55
349	7.56

EP4

Residue number	B-W Numbering
57	2.42
58	2.43
59	2.44
60	2.45
61	2.46
62	2.47
63	2.48
64	2.49
65	2.5
66	2.51
67	2.52
68	2.53
69	2.54

70	2.55
71	2.56
72	2.57
73	2.58
74	2.59
75	2.6
76	2.61
77	2.62
78	2.63
79	2.64
80	2.65
81	2.66
82	2.67
88	3.21
89	3.22

90	3.23
91	3.24
92	3.25
93	3.26
94	3.27
95	3.28
96	3.29
97	3.3
98	3.31
99	3.32
100	3.33
101	3.34
102	3.35
103	3.36
104	3.37

105	3.38
106	3.39
107	3.4
108	3.41
109	3.42
110	3.43
111	3.44
112	3.45
113	3.46
114	3.47
115	3.48
116	3.49
117	3.5
118	3.51
119	3.52

120	3.53
121	3.54
133	4.39
134	4.4
135	4.41
136	4.42
137	4.43
138	4.44
139	4.45
140	4.46
141	4.47
142	4.48
143	4.49
144	4.5
145	4.51
146	4.52
147	4.53
148	4.54
149	4.55
150	4.56
151	4.57
181	5.35
182	5.36
183	5.37
184	5.38
185	5.39
186	5.4
187	5.41
188	5.42
189	5.43

190	5.44
191	5.45
192	5.46
193	5.47
194	5.48
195	5.49
196	5.5
197	5.51
198	5.52
199	5.53
200	5.54
201	5.55
202	5.56
203	5.57
204	5.58
205	5.59
206	5.6
266	6.29
267	6.3
268	6.31
269	6.32
270	6.33
271	6.34
272	6.35
273	6.36
274	6.37
275	6.38
276	6.39
277	6.4
278	6.41

279	6.42
280	6.43
281	6.44
282	6.45
283	6.46
284	6.47
285	6.48
286	6.49
287	6.5
288	6.51
289	6.52
290	6.53
291	6.54
292	6.55
293	6.56
294	6.57
295	6.58
296	6.59
297	6.6
312	7.36
313	7.37
314	7.38
315	7.39
316	7.4
317	7.41
318	7.42
319	7.43
320	7.44
321	7.45
322	7.46

323	7.47
324	7.48
325	7.49
326	7.5
327	7.51
328	7.52
329	7.53
330	7.54
331	7.55
332	7.56
